# Trial participants’ self-reported understanding of randomisation phrases in participation information leaflets can be high, but acceptability of some descriptions is low, especially those linked to gambling and luck

**DOI:** 10.1186/s13063-024-08217-3

**Published:** 2024-06-18

**Authors:** Frances Shiely, Ellen Murphy, Katie Gilles, Kerry Hood, Lydia O’Sullivan, Nicola Harman, Talia Isaacs, Shaun Treweek

**Affiliations:** 1 TRAMS (Trials Research and Methodologies Unit), HRB Clinical Research Facility, University College Cork, Cork, Ireland; 2 Health Research Board Trial Methodology Research Network (HRB TMRN), University College Cork, Cork, Ireland; 3 School of Public Health, University College Cork, Cork, Ireland; 4 Health Services Research Unit, University of Aberdeen, Aberdeen, UK; 5 Centre for Trials Research, Cardiff University, Cardiff, UK; 6 Department of Health Data Science, University of Liverpool, Liverpool, UK; 7 IOE, Faculty of Education and Society, University College London, London, UK

**Keywords:** Randomisation, Trials methodology, Randomised controlled trial, Participant information leaflets, Informed consent

## Abstract

**Background:**

Evidence indicates that trial participants often struggle to understand participant information leaflets (PILs) for clinical trials, including the concept of randomisation. We analysed the language used to describe randomisation in PILs and determine the most understandable and acceptable description through public and participant feedback.

**Methods:**

We collected 280 PILs/informed consent forms and one video animation from clinical research facilities/clinical trial units in Ireland and the UK. We extracted text on how randomisation was described, plus trial characteristics. We conducted content analysis to group the randomisation phrases inductively. We then excluded phrases that appeared more than once or were very similar to others. The final list of randomisation phrases was then presented to an online panel of participants and the public. Panel members were asked to rate each phrase on a 5-point Likert scale in terms of their understanding of the phrase, confidence in their understanding and acceptability of the phrase.

**Results:**

Two hundred and eighty PILs and the transcribed text from one video animation represented 229 ongoing or concluded trials. The pragmatic content analysis generated five inductive categories: (1) explanation of why randomisation is required in trials; (2) synonyms for randomisation; (3) comparative randomisation phrases; (4) elaborative phrases for randomisation (5) and phrases that describe the process of randomisation. We had 48 unique phrases, which were shared with 73 participants and members of the public. Phrases that were well understood were not necessarily acceptable. Participants understood, but disliked, comparative phrases that referenced gambling, e.g. toss of a coin, like a lottery, roll of a die. They also disliked phrases that attributed decision-making to computers or automated systems. Participants liked plain language descriptions of what randomisation is and those that did not use comparative phrases.

**Conclusions:**

Potential trial participants are clear on their likes and dislikes when it comes to describing randomisation in PILs. We make five recommendations for practice.

**Supplementary Information:**

The online version contains supplementary material available at 10.1186/s13063-024-08217-3.

## Background

Informed consent is a regulatory requirement when recruiting participants to clinical trials [1]. It is vital that participant information leaflets (PILs) and informed consent forms (ICFs) provide potential trial participants with information that enables an informed, autonomous decision regarding trial participation [2]. One of the five key aspects of informed consent is understanding [3], meaning potential participants are able to adequately comprehend the information that is provided to them about the trial, in whatever format e.g. written, verbal, audio-visual, etc. Essential information provided includes the following; the trial purpose, the methods of data collection, potential risks, benefits [4], as well as freedom to withdraw from the study at any time without any impact on current treatment [5]. Despite the importance of ensuring consent is informed, evidence shows that participants often have difficulty understanding the information that is provided in trial PILs/ICFs [4–6].

We also know that key trial concepts are not well understood by potential participants [6–15] and many studies have demonstrated that ‘randomisation’ in particular is poorly understood [3, 6–17]. Participants often have a basic understanding of randomisation through analogies, such as picking names out of a hat, but few fully understand randomisation and its purpose in trials [10]. Difficulty understanding the concept of randomisation was identified more than 25 years ago [18], and evidence today tells us that nothing has changed. If the concept of randomisation is not well understood by trial participants, it may lead to difficulty distinguishing between standard care and the intervention, leading to ‘therapeutic misconception’ [10, 19, 20]. A recent review of PILs and ICFs used in Ireland and the UK found that PILs and ICFs are ‘inappropriately complex’ when evaluated against both traditional readability criteria and health literacy-based tools [2, 21].

There is also debate about the need for participants to understand the concept of randomisation or if it is enough to say that allocation to treatment cannot be based on need in a trial [22]. However, custom and practice is to include a description of randomisation in PILs. Therefore, the challenge facing trialists is how to provide this information in a way that potential participants can understand and use to make an informed decision about participation, whilst simultaneously adhering to ethical and regulatory guidance [2]. Our study thus had two goals: (1) to examine the language used to describe the randomisation process in a sample of PILs and ICFs used in Ireland and the UK and (2) to present the randomisation descriptions to participants and the public to determine which descriptions of randomisation are best understood and most acceptable to them.

## Methods

### Data collection

We contacted clinical research facilities/clinical trial units (CRFs/CTUs) in Ireland and the UK to provide us with PIL/ICFs and other available materials used when recruiting participants to randomised controlled trials (RCTs). The study team provided PILs with an agreement to sharing them in a PIL repository where they were able to do so. We collected PILs, ICFs (often integrated with the PIL if provided) and video animations.

Our inclusion criteria were as follows: RCTs at any phase; cohort studies that had an RCT embedded; feasibility and pilot studies; any language. We included adult PILs, PILs provided to parents whose children were being recruited to a trial, PILs for legal representatives/family members of participants who did not have capacity to consent, PILs for adults who regained capacity during the trial and PILs for children. Some studies had a separate PIL and ICF, and where the information was duplicated, we only included the PIL. Integrated PIL/ICFs were included but only information from the PIL was analysed, as the randomisation information was duplicated in the ICF in all cases. We excluded PILs from studies that did not provide a randomisation explanation, e.g. biobank and tissue sample studies. Only the main RCT study documents were included; sub-study documents were excluded.

PILs of trials for vulnerable populations were also included. Vulnerable populations were defined as per the ethics committee definition at University College Cork, Ireland and ICH-GCP definition [20] and included infants and children aged 17 years and under, pregnant women, institutionalised individuals (prisoners, in nursing homes, mental health institutions), critically ill/intensive care unit patients/patients on ventilators unable to provide consent, adults aged 60 and over, participants with learning disabilities, adults with dementia, adults with terminal illness, homeless individuals and refugees, adults with mental illness and members of the armed forces and medical/nursing/dental/pharmacy students where there is a hierarchy that may influence the decision to take part voluntarily.

### Data extraction

PILs were reviewed and analysed for content relating to the description of ‘randomisation’ and other information on trial characteristics outlined in Table 1.

**Table 1:** Data extracted from PILs

• Name of the trial • Study design • Phase of the trial • Commercial or non-commercial trial • Organisation that approved the PIL • PIL version and date • Study population • If the study population was vulnerable • Disease area • Intervention and comparator • Drug/non-drug/mix of intervention types • If it was the main PIL • Were additional documents provided • Permission for the PIL to be uploaded to a public repository • PPI involvement • Inclusion of graphic images • The language of the PIL • Was it an integrated PIL and ICF
**Data specific to randomisation**
• Explanation of the randomisation process • The level of randomisation (cluster or individual) • The randomisation ratio • Non-text supporting materials that depict randomisation

Where two or more PILs were provided for a single trial, we included the most recent version of the document. If it was unclear which PIL was used, information from both was used. Similarly, if two or more PILs were provided for the same trial that were intended for distribution in different locations, e.g. Wales, Scotland, and England, but the wording on randomisation was the same, only one PIL was selected for analysis.

The information to be extracted from the documents was discussed and agreed upon by all members of the research team. The data extraction was conducted independently by EM and LOS on separate groups of PILs and transferred to a single Microsoft Excel file. Any uncertainties regarding the classification of trial characteristics were discussed and agreed upon by EM and FS and where required by consensus at a team meeting consisting of all authors. The video included an audio description of randomisation that was transcribed, and the content was analysed together with the other written PILs.

### Qualitative analysis—pragmatic content analysis

Analysis of the description of randomisation was conducted using pragmatic content analysis as outlined by Bengtsson [23]. There are four stages: decontextualisation, recontextualisation, categorisation and compilation [23]. The unit of analysis was the PIL. The ‘meaning unit’, as outlined by Bengtsson, is the smallest unit that contains the information the researcher needs [23]. The meaning units in our study were defined as words, phrases and sentences that were associated with the process of randomisation, for example, ‘The particular treatment given to each person in the study will be decided by computer allocation. If you decide to take part in this study, this will mean that neither you nor your doctors can decide which treatment you will receive. There is an equal chance you will be placed into either treatment group’. We considered this to be a meaning unit.

#### Stage 1—De-contextualisation

All PILs were reviewed, and the meaning units were extracted. The meaning units were coded using colour-coded text in Microsoft Excel. The codes were created inductively during the de-contextualisation process as more PILs were reviewed. The data were coded by one researcher, EM, and the coding was reviewed by FS to increase the reliability of the coding framework. To further increase the reliability of the coding framework, the first 40 PILs/ICFs were reviewed by KG to ensure consistency of the coding [24].

#### Stage 2—Re-contextualisation

The meaning units and codes were reviewed and re-read with the original information from the PILs to ensure the information was captured sufficiently. Any information that was not relevant to the description of the concept of randomisation was excluded.

#### Stage 3—Categorisation

The codes were analysed and grouped into categories and sub-categories. This was done in a discursive online meeting with EM, FS and KG.

#### Stage 4—Compilation

Manifest analysis guided the compilation. This means the original text was referred to, to ensure the categories and sub-categories stayed close to the information provided in the PILs. The themes were narratively described and were also quantified where appropriate.

### Consensus meeting

All authors met to discuss the analysis and formulate a questionnaire to be sent for public consultation. All authors agreed on the categories and sub-categories proposed by EM, FS and KG (Stage 3—Categorisation). We excluded phrases that appeared more than once or were similar in meaning. We compiled these phrases in an online questionnaire to send to potential trial participants and members of the public.

### Public involvement consultation

A copy of the questionnaire distributed for consultation is available in Additional file 1. We distributed the questionnaire via our own networks by email, HRB TMRN (HRB Trials Methodology Research Network, Ireland), MRC-NIHR-TMRP (Medical Research Council—National Institute for Health and Care Research—Trial Methodology Research Partnership, UK, UKTMN (UK Trial Managers Network), and via our own existing research collaborations and by Twitter. The membership of these networks is broad and includes people working in trials, trial managers and patient and public involvement members. We asked these colleagues to distribute the questionnaire to their PPI contacts and colleagues. We addressed the questionnaire as ‘Dear PPI colleague’ to ensure only patients and members of the public filled it in. We asked participants to read each randomisation statement and to rate each statement on a five-point Likert scale (Very poor, Poor, Neutral, Good, Very good) in terms of (a) their understanding of the statement, (b) their confidence in their understanding of the statement and (c) the acceptability of the statement. Seventy-three participants completed the questionnaire. Participants were able to leave additional comments after each section about the phrases within the section, if desired. We summarised these narratively.

### Quantitative analysis

Descriptive statistics were produced with frequencies and proportions reported.

## Results

### Sample characteristics

We received and analysed 280 paper-based PILs and the transcribed text from 1 video animation. All were in the English language. Together this represented 229 trials, either ongoing or concluded, in Ireland and the UK. Table 2 displays the trial and PIL characteristics.

**Table 2:** Trial and participant information leaflet characteristics

**Trial characteristics**	**Number of trials (*n* = 229)**
**Non-drug trials**^**a**^	102
**Drug trials**^**a**^	49
**Mixed trials**^**a**^	71
**Vaccine trials**	2
**Unsure**	4
**No information**	1
**2-arm trials**	191
**3-arm trials**	21
**4-arm trials**	6
**5-arm trials**	3
**6-arm trials**	1
**Other**^**b**^	6
**Unclear**	1
**Commercial**^**c**^	41
**Non-commercial**	183
**No information**	5
**Trials involving vulnerable populations**	72
**Trials with multiple PILs**	22
**Disease area (defined by NIHR specialty area)**	**Number of trials**
Cancer	34
Cardiovascular disease	24
Reproductive health	22
Gastroenterology	19
Kidney and urinary tract	19
Respiratory disorders	11
Children and young people	8
Dementias and neurodegeneration	7
Trauma and emergency care	7
Endocrinology	7
Mental health	5
Infectious diseases	5
Musculoskeletal disorders	5
COVID	5
Neurological disorder	5
Dermatology	4
Anaesthesia, perioperative medicine and pain management	4
Critical care	3
Oral and dental health	3
Surgery	2
Physiotherapy	2
Other^e^	28
**PIL characteristics**	**Number of PILs (*n* = 280)**
Mention of PPI involvement	19 PILs
Graphics of any sort in the PIL^d^	96 PILs plus the video animation
PILs developed for children	38 PILs
PILs developed for parents	32 PILs
PILs developed for legal representatives	9 PILs
PILs for develop for those that recover capacity	14 PILs

^a^Drug trials were classified as trials where both the intervention and comparator were drugs. Non-drug trials were classified as trials where both the intervention and comparator were non-drug. Mixed trials were classified as trials where the intervention or comparator were drug or non-drug

^b^These trials included, for example depending on the severity of illness, different numbers of intervention options available to participants

^c^Commercial trials were classified as trials where there is funding from a commercial company or if there were links to commercial companies such as drug manufacturers that could potentially cause conflicts of interest, e.g. drug companies providing the trial with drugs free of charge. Non-commercial trials were trials that were funded via government funding or charity and had no funding links to commercial companies

^d^Graphics include tables, flow charts and images of any description

^e^Other disease/non-disease areas where singular clinical trials were conducted

### Descriptions of randomisation

Table 3 summarises the descriptions of randomisation. Of 280 PILs, 21 (7.5%) PILs did not include any explicit explanation of the term ‘randomisation’, and ten of these PILs were for children. Only 11 PILs (3.9%) and the video animation contained a non-text description of randomisation, e.g. an image of a dice, an image of a computer and two groups of people, etc.

**Table 3:** Randomisation descriptions

Randomisation information	Number of trials (*n* = 229)/PILs (*n* = 280)
Individual level randomisation	224 trials (97.8%)
Cluster level randomisation	5 trials (2.2%)
PILs with no explanation of randomisation	21 PILs (9.17%)
PILs with non-text supporting material to describe randomisation	11 PILs plus 1 video animation (4.3%)
**Randomisation ratio**	
Randomisation ratio 1:1	1 PIL (0.4%)
Randomisation ratio indicated to be 1:1	184 PILs plus 1 video animation (66.1%)
Randomisation ratio not 1:1	5 PILs (1.8%)
Randomisation ratio unclear	90 PILs (32.1%)

Regarding the trial randomisation ratio, only 1 PIL contained an explicit description of the randomisation ratio the trial was using ‘ratio 1:1:1 – so you will have a 33.3% chance of receiving either dose’. For 90 PILs, it was unclear whether the ratio was 1:1 or not. In 184 PILs and the video animation, there was information that indicated the randomisation ratio was 1:1. By indicated we mean the PIL contained phrases such as ‘allocation to these groups is random like tossing a coin’, ‘you will have an equal chance of being allocated to either group’, ‘one half of the participants will be assigned to receive treatment,’ ‘and the other half assigned to receive placebo’ and ‘you have an equal chance of being assigned to one of the 4 treatment groups’. Five PILs had a randomisation ratio that was not 1:1, and in these five, all explained the ratio with examples, e.g. ‘This study has been designed so that there is more chance of a baby receiving the alcohol-based antiseptic (3:1 ratio)’ and ‘Two-thirds of patients involved will be allocated to the new treatment group, one-third will be allocated to the ‘usual care alone’ group’.

### Pragmatic content analysis

The pragmatic content analysis resulted in five inductive categories and 48 randomisation phrases (Table 4).

**Table 4:** Inductive categories of randomisation

Category	Theme	Number (*n* = 48)
1	Explanation of why randomisation is required in clinical trials	10
2	Randomisation synonyms (phrases used to describe randomisation—using different words that mean randomisation)	9
3	Comparative phrases (phrases that compare randomisation to something else)	11
4	Elaborating phrases (phrases that give further details of the randomisation)	7
5	Phrases that describe the process of randomisation	11

### Ratings of the randomisation phrases

Participants rated each of the 48 randomisation phrases on a five-point Likert scale: ‘Very poor’, ‘Poor’, ‘Neutral’, ‘Good’, ‘Very good’. They rated ‘My understanding of this statement is…’, ‘My confidence in my understanding of this statement is…’ and ‘I think the acceptability of this statement is…’. We combined the proportions for the ‘Very good’ and ‘Good’ categories for each of the three ratings a posteriori. We ranked each randomisation phrase according to the response to, ‘I think the acceptability of this [*randomisation*] statement is…’ to determine the top five ranking phrases according to acceptability. We took the same approach for ‘Very poor’ and ‘Poor’ to determine the lowest five ranking phrases according to acceptability.

The inductive categories, summaries of the text of the frequently used examples (paraphrased and merged text to summarise the category in lay terms), a sample quote from a PIL, and the maximum and minimum acceptability scores for each of the five inductive categories, are presented in Table 5.

**Table 5:** Frequency of phrases included in the PILs

Randomisation category	Summary of descriptions	Sample quote	PILs (*n* = 281) and video animation (*n* = 1)	Minimum acceptability score for category	Maximum acceptability score for category
**1. Explanation of why randomisation is required in trials**	To ensure that a fair comparison can be made between the groups and/or that bias is avoided	‘This is essential so that a fair comparison can be made between the two groups. Dividing people into treatment groups in this way is what is called a ‘randomised clinical trial’ and is the standard and most reliable way of comparing different treatment options’	91 (32.4%)	45.2	80.8
	To ensure the groups are the same at the start of the trial	‘to ensure that people in each group are equally matched’	29 (10.3%)		
**2. Synonyms for randomisation**	Other words, e.g. you will be randomly assigned, you will be allocated at random, allocated by a process of randomisation	‘You will be randomly assigned to one of 2 groups’	65 (23.1%)	39.5	71.2
**3. Comparative randomisation phrases**	Comparing randomisation to the toss of a coin, or roll of a die, or lottery	‘…using a process which is similar to tossing a coin’	93 (33.1%)	24.7	69.8
**4. Elaborative phrases further explaining randomisation**	Explaining that there is an equal chance of being allocated to each of the groups in the trial, or that half of participants would receive one treatment and the other half would receive another	‘Your child will have the same chance of being put in either group’	167 (59.4%)	50.7	78.1
**5. Phrases that describe the process of randomisation**	Explaining randomisation was carried out by a computer, or centrally in a study office, or using sealed envelopes	‘…treatment given to each person in the study will be decided by a computer allocation. this will mean that neither you nor your doctors can decide which treatment you will receive’	135 (48%)	24.6	79.5
	Explaining that the participant/their doctor or any other person involved in the trial was not able to choose the participants treatment	‘neither you nor your doctors can decide which treatment you will receive’	89 (31.8%)		
**No explanation of randomisation**			21 (7.5%)		

### Acceptability of phrases in the five randomisation categories

Seventy-three people responded to our questionnaire to rate their understanding of, confidence in their understanding of and acceptability of, 48 phrases describing randomisation. All 48 phrases are presented in Additional file 1. Of the 48 phrases, only 11 were above 70% in terms of acceptability. The top five phrases in terms of acceptability (proportion of people rating them ‘Very good/Good’ on a 5-point Likert scale) and the lowest ranked in terms of acceptability (proportion of people rating them ‘Very poor/Poor’ on a five-point Likert scale) are presented in Tables 6 and 7 respectively. For the top five phrases, understanding of and confidence of understanding of all phrases is high. The most notable variation is in terms of acceptability. Two of the top five phrases came from category 1 ‘Explanation of why randomisation is required’. In terms of phrases that ranked low on acceptability, two were from category 5, two from category 3 and one from category 2. Additional file 2 contains the mean scores for each inductive category. Category 4, ‘Elaborating phrases further explaining randomisation’, had the highest mean acceptability score (68.2) along with the highest mean understanding score (84). Category 5, phrases that described the process of randomisation, had the lowest mean score (49.2) as well as the lowest mean understanding score (72.6), followed closely by category 3, comparative phrases, 49.9 and 75.8 respectively. A table of the top 10 ranked phrases can be found in Additional file 3.

**Table 6:** Top five acceptable randomisation phrases (‘Very good/Good’ combined)

Top 5 phrases	Phrase with high acceptability	Inductive category to which phrase belongs	Understanding (%)	Confidence in understanding (%)	Acceptability (%)
1	**‘We do randomised trials when there is more than one treatment option available for patients with a disease and we don’t know which one is best. In order to find out, we need to compare the different treatments. So we put people into groups and give each group a different treatment. The results from the different treatment groups are compared to see if one treatment is better. To try to make sure the groups are the same to start with, each patient is put into a group by chance (randomly).’**	Category 1: Why randomisation required	90.4	87.6	80.8
2	**‘Neither you nor your clinical team will be able to decide which study treatment you receive.’**	Category 5: Process of randomisation	90.4	89	79.5
3	**‘There is an equal (50:50) chance of being allocated to one group or the other’**	Category 4: Elaborating randomisation phrases	90.4	86.3	78.1
4	**‘When we do not know which way of treating patients is best, we need to make a comparison. An important part of making a fair comparison is “randomisation”. Most large trials are randomised. Patients taking part are randomly allocated either the standard treatment or the research treatment. This process is essential to avoid bias: if the groups receiving each treatment are the same, any differences in the results can only be down to the treatments. Therefore, randomisation means that the results are more reliable.’**	Category 1: Why randomisation required	91.8	89	75.3
5	**‘You will have a 50% chance of being in either group’**	Category 4: Elaborating randomisation phrases	89	87.7	74

**Table 7:** Top five unacceptable randomisation phrases ('Very poor/Poor' combined)

	**Phrase with low acceptability**	**Inductive category to which phrase belongs**	Understanding* (%)	**Confidence in understanding**^**a**^** (%)**	**Acceptability rating (%)**
1	**‘By taking part in the study you will give up your right to choose which treatment you receive.’**	Category 5: Phrases that describe the process of randomisation	17.8	23.3	57.5
2	**‘If you decide to join the trial, you may receive the investigational treatment chosen by chance, as if on the roll of a dice’**	Category 3: Comparative phrases	21.9	23.3	50.7
3	**‘That the [group] you (are invited to) join is determined by a sophisticated machine designed for this purpose, and not influenced by us’**	Category 5: Phrases that describe the process of randomisation	20.5	26	41.1
4	**‘The choice of what to give (active treatment or dummy treatment) is made randomly (like a lottery)’**	Category 3: Comparative phrases	13.7	17.8	42.5
5	**‘The type of medication you get will be decided at random.’**	Category 2: Randomisation synonyms—using different words that mean randomisation	15	16.4	39.7

^a^The lower the rating, the higher the understanding

### Additional comments from participants on each of the five categories

Additional comments for each category were given, and we describe them narratively here. In category 1—explanation of why randomisation is required in clinical trials—37/73 participants left additional comments. There were conflicting views in terms of sentence length, with some asking to ‘keep it simple', or ‘short’ and others saying they find the longer explanations more useful. However, the clear message was trialists should keep the language ‘simple'…use 'plain English'…and 'avoid repetition’. Of note, two people made it clear that they rated the acceptability in terms of how others would interpret it. They felt their own understanding was very good, but others might have difficulty.

In category 2—randomisation synonyms—31 people left additional comments. Similar to category 1, participants wanted to keep the language simple. Furthermore, individuals emphasized the importance of inclusivity and requested that we avoid assuming that individuals who do not speak English as their primary language will comprehend the term ‘random.’

In category 3—comparative phrases—35 participants gave additional comments. Responses were uniform with an overwhelming emphasis on the dislike for the analogy with gambling, or luck:

- ‘The above statements are a shocking way to let patients know about part of the trial. Very insensitive!!’
- ‘Reference to a lottery or coin tossing may well put people off taking part’
- ‘Most of us never won anything so this whole randomisation will automatically be associated with failure’

Participants also felt that using terms such as flipping a coin and rolling a dice were not interchangeable and did not portray an equal chance of group allocation. In addition, participants highlighted that these analogies were not suitable for under-served groups or individuals whose first language is not English;

- ‘English is not my first language and I did not know the meaning of dice’
- ‘Do you need to consider minorities who may find gambling abhorrent? Against their religion’

In category 4—elaborating phrases—26 people provided additional comments, mostly relating to the mathematical ability necessary when describing randomisation. The primary concern was the mathematical literacy required to understand proportions and ratios:

- ‘Consent is not a maths test’
- ‘Try not to use numbers. They give many people nightmares’
- ‘50/50 and % can be less well understood by many’

In category 5—phrases that describe the process of randomisation—30 participants left additional comments. There was a focus on the use of sealed envelopes for randomisation, with some saying it’s:

- ‘Outdated’
- ‘Is it true that this is how randomisation is done?’
- ‘Statement 56…although useful as illustration, is that ACTUALLY how they do randomisation’

Others were uncomfortable with the use of the phrase ‘sophisticated machine’ stating itis patronising and childlike and they did not like the idea that the machine chooses the treatment:

- ‘Computer programme or just Computer would be a simple explanation’
- ‘I think older people may be unsure/ hesitant about taking part if it says that computers are choosing which treatment they get’
- ‘Statements that mention giving up rights should be absolutely avoided’

## Discussion

Randomised trials are one of the most important elements of an evidence-based health care system. All of them depend on the willingness of patients and the public to take part in them, and participant information leaflets (PILs) are one of the ways in which potential participants are told about the trial, including that it will involve randomisation. In our study of 229 PILs from Ireland and the UK, including 259 randomisation phrases, we have found variation in how understandable and, especially, how acceptable some phrases about randomisation are to potential participants. In particular, trialists’ frequent recourse to gambling analogies to describe randomisation leaves potential participants feeling cold; the analogies are understood but unacceptable. Remarkably, four of the five most acceptable phrases come from categories 1 (why randomisation is required) and 4 (elaborating randomisation phrases); thus, participants are telling us clearly they want to know in detail why randomisation is required, rather than how it is conducted.

Earlier studies have found, unlike us, that participants find it difficult to understand randomisation [3, 6–17]. We found that the proportion of participants rating their understanding of randomisation phrases as ‘Very good/Good’ on a five-point Likert scale had a mean of more than 70% for each of the five inductive randomisation categories. We also found that phrases that were unacceptable had a high understanding. Looking in more depth at the phrases that had the lowest acceptability, a pattern emerges; all five have a common theme, i.e. the removal of control, or loss of control, over the trial process, e.g. ‘By taking part in the study you will give up your right to choose which treatment you receive’, ‘That the [group] you (are invited to) join is determined by a sophisticated machine designed for this purpose, and not influenced by us’, ‘The choice of what to give (active treatment or dummy treatment) is made randomly (like a lottery)’.

Our study is consistent with a prior qualitative study of audio recordings of recruitment consultations [20] where the authors reported that it was a problem when a computer had agency over how allocation was made, e.g. ‘The treatment which you receive will be picked by a computer which has no information about you, that is, by chance’ rated 77% for understanding and 45% for acceptability. This suggests that the language, structure and clarity used to explain the process of randomisation needs revisiting to ensure better participant engagement and comprehension. A similar finding in an embedded qualitative study within a trial found that families being recruited to a trial for conservative treatment for appendicitis versus surgery were confused by the use of the computer in the randomisation process, incorrectly thinking the computer selected the treatment most appropriate for the child [25].

Our findings should also be compared with two older cancer studies. A 2002 study [17] provided the public, patients and cancer physicians with seven contrived randomisation phrases, all mentioning either computers or chance. The authors concede that it was difficult to identify ‘the best’ way to describe the process of randomisation because the proportions choosing the statements were so similar. Likewise, in a 2005 study [26] by the same authors, seven randomisation phrases were reported, taken from PILs. Over 100 definitions of randomisation were condensed to seven, without explaining the process. Like the previous study, all seven phrases mentioned computers, chance, or both. In both studies, the respondents did not have a choice to reject the use of phrases that included computers and chance. In our study, they did have that opportunity due to the larger number and variety of phrases. Thus, given our findings, taken with Jepson’s [20] findings, we recommend that reference to computers or machines making randomisation decisions is avoided where possible in future PILs.

At the opposite end of the spectrum, our analysis revealed that phrases explaining why randomisation is required (category 1) and phrases elaborating on randomisation (category 4) exhibited the highest levels of acceptability and understanding among the participants, surpassing other categories by a significant margin. This suggests that participants found these categories more accessible, comprehensible and relatable and found the explanations in these categories to be effective in conveying the underlying principles and procedures of randomisation. The additional comments provided tell us that participants want the language to be simple, without numbers, and written in plain English. We know that this is not often achieved in PILs [2] or trial reports [27]. Our previous study on the readability of trial lay summaries, which should meet the information needs of the general public, found that none of the 60 trial lay summaries analysed met the recommended reading age of 11–12 years for health literature and 85% were considered difficult to read [27]. We reiterate our recommendations made in that study: use the freely available webtool (https://www.webfx.com/tools/read-able/#enter-text-tab) when preparing trial related information to establish their readability on the SMOG (Simple Measure of Gobbledygook) scale and use the SMOG scale in conjunction with plain language guidelines for each language in which the PIL will be disseminated. Key to ensuring accessibility is involving trial participants, patients and the public (PPI) in the planning, development, translation and dissemination of PILs.

One third of PILs in our sample used comparative phrases (category 3), like comparing randomisation to a lottery, coin toss or die roll. These phrases, along with those describing the process of randomisation (category 5), received the lowest acceptability scores. Participants strongly disliked gambling or luck analogies for randomisation, finding them unsettling and potentially discouraging for potential participants. This sentiment aligns with a previous qualitative study of 73 recruitment consultations [20] where concerns were raised about the insensitivity of such analogies in conveying crucial trial information.

Participants in our study also noted the unsuitability of these analogies for some under-served groups or individuals for whom English is not their first language. They emphasized that ‘tossing a coin and rolling dice may be understood by native English speakers but may be less well understood by those who have English as a second language’. This highlights the importance of considering inclusivity and ensuring that language used in participant materials is accessible to diverse populations. In addition to concerns about understanding and inclusivity, participants raised questions about the compatibility of these analogies with certain religious beliefs or cultural backgrounds. They pointed out that some minority groups may find the reference to gambling abhorrent or contradictory to their religious and other values and principles, and this may create barriers to their participation. These comments provide valuable insights into the perception and concerns of potential participants regarding the use of gambling-related analogies in describing randomisation. In fact, none of the comparative phrases (category 3) achieved an acceptability in excess of 70%. Thus, we recommend avoiding comparative phrases when describing randomisation in PILs, especially those linked to gambling and luck, and focusing on what participants found acceptable when describing randomisation, i.e. why randomisation is required (category 1), elaborating randomisation phrases (category 4) and the process of randomisation (category 5). One means of ensuring the most appropriate randomisation phrases are used for the intended population is to design the PIL with PPI (there are many PPI groups in CRF/CTUs), but it would be important to ensure that those from the intervention target group are included in that design process. Additionally, piloting the PIL in advance of initial recruitment on a small sample of those from the target population would be beneficial to provide feedback on understanding and acceptability.

Our participants have voiced their concerns and preferences in this study, and we have provided examples of randomisation phrases that are sensitive, inclusive and easily understandable across diverse populations. By addressing these concerns, researchers can enhance the informed consent process and facilitate more meaningful participation in clinical trials.

## Strengths and limitations

One of the key strengths of this paper is the drawing together of so many different PIL to explore the variation in randomisation descriptions (280 PILs, from more than 24 CTUs/CRFs contributed 259 randomisation phrases). These included drug, non-drug and vaccine trials, commercial and academic trials, varying trial populations, varying disease areas, a variety of trial design and PILs developed for children, parents, legal representatives and those that recover capacity. The consultation with the public is also a strength. However, there are some limitations. Though we advertised for patients and the public to fill in the questionnaire and addressed our questionnaire to PPI colleagues, we have no way of knowing if others in the trial community through the networks which we distributed the questionnaires filled it in. This is a limitation of our study. This study focused solely on the PIL and the randomisation text within. We did not consider the conversation that takes place between recruiter and potential trial participantd during the informed consent process. This could be considered a limitation, but this work has already been done by Jepson and colleagues [20], and we did not seek to repeat it here. Rather, a strength of this study is that it has confirmed the Jepson findings, and we can definitively make recommendations for practice that are supported by both independent studies targeting the two different aspects of the informed consent process. We did not collect the sociodemographic characteristics of the PPI colleagues invited to complete the questionnaire. Thus, our findings may vary across different sociodemographic groups, i.e. ethnicity, socio-economic background, etc., and our results should be interpreted with this in mind.

**Table:** 

**Implications for practice** 1. PILs should exclude any reference to gambling; those recruiting should not use gambling analogies in their explanations 2. We recommend removing any phrases that mention computers or automated systems 3. PILs should avoid comparative phrases (category 3) 4. Those writing PILS should consider the diversity of society and aim for phrases that are understandable and acceptable for everyone 5. CTUs/CRFs tend to use the same PIL templates. Whilst it is okay to begin with an existing PIL template, we recommend modifying it to suit the population, intervention and the intended target group	

## Conclusions

We have shown that potential trial participants know what they like and dislike when describing randomisation in PILs and randomisation phrases that are well understood are not necessarily acceptable. Our participants made a clear statement on the need for randomisation descriptions to be inclusive. Our study, taken together with the Jepson et al. findings [20] provides compelling evidence for avoiding the use of comparative gambling/gaming phrases and those that attribute decision-making to computers or automated systems.

### Supplementary Information

Additional file 1: Randomisation Phrases.

Additional file 2: Acceptability, understanding and confidence in understanding scores for each category of phrases.

Additional file 3: Top 10 randomisation phrases ranked by acceptability.
