# Supplementary material for: Trial participants’ self-reported understanding of randomisation phrases in participation information leaflets can be high, but acceptability of some descriptions is low, especially those linked to gambling and luck
Source: Trials. 2024 Jun 18;25:391. doi: 10.1186/s13063-024-08217-3 (PMC11186130; doi:10.1186/s13063-024-08217-3)
Supplement: Supplementary file 1 — Additional file 1: Randomisation Phrases. [file 13063_2024_8217_MOESM1_ESM.pdf]

EXCELSIOR Phrases

EXploring the ComplExity and Language of ConSent and participation InfOrmation in Randomised Controlled Trials (EXCELSIOR)

Dear PPI colleague,

We need your help to find out how best to describe randomisation to patients and the public. The information that potential clinical trial participants receive typically includes a description of randomisation. Previous research tells us that people may interpret how randomisation works, differently. We are interested in finding the best way to explain randomisation to potential participants to help them make an informed decision on whether or not to participate in the clinical trial.

We have worked through a large body of patient information leaflets from completed trials and we pulled out the phrases used to explain randomisation. We would like you to help us by rating each of the phrases in the following pages by circling the number on the Likert scale (very poor, poor, neutral, good and very good) that best represents your perceived understanding of the phrase, how acceptable you find the phrase to be, and your confidence in your perceived understanding of the phrase. We will use your feedback to write a description of randomisation that is accurate and easily understood by you. We will then share this with other researchers so that they can use it in the future when writing participant information leaflets.

We expect the questionnaire to take you around 30 minutes to complete. The questionnaire is split into five categories.

Category 1: Explanations of why randomisation is required in clinical trials
Category 2: Phrases used to describe randomisation (randomisation synonyms- using different words that mean randomisation)
Category 3: Phrases that compare randomisation to something else (comparative phrases)
Category 4: Phrases that give further details of the randomisation (elaborating phrases)
Category 5: Phrases that describe the process of randomisation

Thank you in advance for helping us with this important project. If you have any questions, please email the study principal investigator, Dr Frances Shiely at f.shiely@ucc.ie.

We would like to give you a €20 Me2You voucher. In order to receive that, please email f.shiely@ucc.ie and I will be delighted to send this on.

The Excelsior Team (Dr Frances Shiely, Ms Ellen Murphy, Professor Shaun Treweek, Dr Katie Gilles, Dr Talia Isaacs, Dr Nicola Harman, Professor Kerry Hood, Ms Elaine Finucane, Professor

Peter Doran, Ms Lydia O'Sullivan)

*Questions marked with a * are required.*

Agreement to Consent to Complete the Questionnaire

I am aware that I am being asked to complete a questionnaire on the topic of "randomisation". I am aware that participation is voluntary and that I may stop at any stage. I am aware that my responses will be anonymous.

The data collected in the study will be kept for a period 10 years for other studies as per UCC policy after the end of the study. Thereafter, they may be stored for a further period of time for legal reasons (e.g. revised retention obligations), or more if required by law.

I understand that if I have any questions, I can contact Dr Frances Shiely (f.shiely@ucc.ie). I understand that the study has been approved by the Clinical Research Ethics Committee of the Cork Teaching Hospitals (CREC) and if I have further queries concerning my rights in connection with the research, I can contact CREC at Lancaster Hall, 6 Little Hanover Street, Cork, 021 4901901.

1. I have read and understand this consent form: *

   ○ Yes

   ○ No

2. I agree to participate and complete the questionnaire: *

   ○ Yes

   ○ No

3. I grant permission for the data collected to be used as part of a research study: *

   ○ Yes

   ○ No

4. I understand that my anonymised data will be stored at University College Cork for up to 10 years: *

   By clicking 'next' I consent to participate in this research project

   ◯ Yes

   ◯ No

Category 1: Explanations of why randomisation is required in clinical trials

5. “This is essential so that a fair comparison can be made between the two groups. Dividing people into treatment groups in this way is what is called a ‘randomised clinical trial’ and is the standard and most reliable way of comparing different treatment options.” *

	Very Poor	Poor	Neutral	Good	Very Good
My understanding of this statement is:	○	○	○	○	○
My confidence in my understanding of this statement is:	○	○	○	○	○
I think the acceptability of this statement is:	○	○	○	○	○

6. “To make sure that we get a true answer from the study we have to ensure that the results will not be biased in any way. In order to achieve this we have to be sure that there is a 50:50 chance of you being treated by either [intervention A] or [intervention B] in the first instance.” *

	Very Poor	Poor	Neutral	Good	Very Good
My understanding of this statement is:	○	○	○	○	○
My confidence in my understanding of this statement is:	○	○	○	○	○
I think the acceptability of this statement is:	○	○	○	○	○

7. “The only way to make sure that the two groups are as similar as possible is to have the treatment decided upon by chance: a process called randomisation. This process ensures that the treatments are compared fully and fairly” *

	Very Poor	Poor	Neutral	Good	Very Good
My understanding of this statement is:	○	○	○	○	○
My confidence in my understanding of this statement is:	○	○	○	○	○
I think the acceptability of this statement is:	○	○	○	○	○

8. “When we do not know which way of treating patients is best, we need to make a comparison. An important part of making a fair comparison is “randomisation”. Most large trials are randomised. Patients taking part are randomly allocated either the standard treatment or the research treatment. This process is essential to avoid bias: if the groups receiving each treatment are the same, any differences in the results can only be down to the treatments. Therefore, randomisation means that the results are more reliable.” *

	Very Poor	Poor	Neutral	Good	Very Good
My understanding of this statement is:	○	○	○	○	○
My confidence in my understanding of this statement is:	○	○	○	○	○
I think the acceptability of this statement is:	○	○	○	○	○

9. “We do randomised trials when there is more than one treatment option available for patients with a disease and we don’t know which one is best. In order to find out, we need to compare the different treatments. So we put people into groups and give each group a different treatment. The results from the different treatment groups are compared to see if one treatment is better. To try to make sure the groups are the same to start with, each patient is put into a group by chance (randomly).” *

	Very Poor	Poor	Neutral	Good	Very Good
My understanding of this statement is:	◯	◯	◯	◯	◯
My confidence in my understanding of this statement is:	◯	◯	◯	◯	◯
I think the acceptability of this statement is:	◯	◯	◯	◯	◯

10. “It is really important that the two groups for the [study name] study have a similar mix of patients in them. Having a similar mix means that we know that if one group of patients does better than the other, it is very likely to be because of the treatment and not because there are differences in the types of patients in each group” *

	Very Poor	Poor	Neutral	Good	Very Good
My understanding of this statement is:	○	○	○	○	○
My confidence in my understanding of this statement is:	○	○	○	○	○
I think the acceptability of this statement is:	○	○	○	○	○

11. “The process of randomisation makes the study scientifically strong and allows the findings to be used to guide treatment of patients in the future.” *

	Very Poor	Poor	Neutral	Good	Very Good
My understanding of this statement is:	○	○	○	○	○
My confidence in my understanding of this statement is:	○	○	○	○	○
I think the acceptability of this statement is:	○	○	○	○	○

12. “This may sound strange but randomisation ensures a fair comparison. The computer programme puts equal numbers of patients of different ages and states of health in each group so that at the end of the study we are sure that any differences between [patients] in the two groups are due to whether or not they had [study intervention], rather than anything else.” *

	Very Poor	Poor	Neutral	Good	Very Good
My understanding of this statement is:	○	○	○	○	○
My confidence in my understanding of this statement is:	○	○	○	○	○
I think the acceptability of this statement is:	○	○	○	○	○

13. “This is the only way we can ensure that the groups are as similar as possible to each other – in age, height, weight, and so on. If you or your doctor choose a treatment, the groups would not be the same” *

	Very Poor	Poor	Neutral	Good	Very Good
My understanding of this statement is:	○	○	○	○	○
My confidence in my understanding of this statement is:	○	○	○	○	○
I think the acceptability of this statement is:	○	○	○	○	○

14. “At the moment we do not know if [treatment] is the best way to improve detection so we need to randomly assign people.... We will then be able to compare what happens between the two groups. The goal of randomisation is to produce comparable groups in terms of general participant characteristics, such as age or gender, and other key factors that affect the probable course a disease would take. In this way, the two groups are as similar as possible at the start of the study.” *

	Very Poor	Poor	Neutral	Good	Very Good
My understanding of this statement is:	○	○	○	○	○
My confidence in my understanding of this statement is:	○	○	○	○	○
I think the acceptability of this statement is:	○	○	○	○	○

15. Do you have any other comments you would like to add?

[ ]

Category 2: Phrases used to describe randomisation

(randomisation synonyms - using different words that mean randomisation)

16. “The study is a randomised trial which means you will be randomly allocated to one of two groups” *

	Very Poor	Poor	Neutral	Good	Very Good
My understanding of this statement is:	○	○	○	○	○
My confidence in my understanding of this statement is:	○	○	○	○	○
I think the acceptability of this statement is:	○	○	○	○	○

17. “Each patient is put into a group by chance (randomly)” *

	Very Poor	Poor	Neutral	Good	Very Good
My understanding of this statement is:	○	○	○	○	○
My confidence in my understanding of this statement is:	○	○	○	○	○
I think the acceptability of this statement is:	○	○	○	○	○

18. “We will then randomly allocate you to either [study drug] or placebo capsules.” *

	Very Poor	Poor	Neutral	Good	Very Good
My understanding of this statement is:	○	○	○	○	○
My confidence in my understanding of this statement is:	○	○	○	○	○
I think the acceptability of this statement is:	○	○	○	○	○

19. “The decision of which of the two treatments you receive will be made by chance (this is called ‘randomisation’).” *

	Very Poor	Poor	Neutral	Good	Very Good
My understanding of this statement is:	○	○	○	○	○
My confidence in my understanding of this statement is:	○	○	○	○	○
I think the acceptability of this statement is:	○	○	○	○	○

20. “Everyone who agrees to take part in this research study will be allocated at random to one of two groups” *

	Very Poor	Poor	Neutral	Good	Very Good
My understanding of this statement is:	○	○	○	○	○
My confidence in my understanding of this statement is:	○	○	○	○	○
I think the acceptability of this statement is:	○	○	○	○	○

21. “The group you will be in will be chosen by chance” *

	Very Poor	Poor	Neutral	Good	Very Good
My understanding of this statement is:	◯	◯	◯	◯	◯
My confidence in my understanding of this statement is:	◯	◯	◯	◯	◯
I think the acceptability of this statement is:	◯	◯	◯	◯	◯

22. “The study which you are being asked to participate in will be randomised which means that you will be allocated at random to receive [placebo] or [study drug].” *

	Very Poor	Poor	Neutral	Good	Very Good
My understanding of this statement is:	○	○	○	○	○
My confidence in my understanding of this statement is:	○	○	○	○	○
I think the acceptability of this statement is:	○	○	○	○	○

23. “The assignment of participants to the two groups will be processed automatically and completely at random.” *

	Very Poor	Poor	Neutral	Good	Very Good
My understanding of this statement is:	○	○	○	○	○
My confidence in my understanding of this statement is:	○	○	○	○	○
I think the acceptability of this statement is:	○	○	○	○	○

24. “The type of medication you get will be decided at random” *

	Very Poor	Poor	Neutral	Good	Very Good
My understanding of this statement is:	◯	◯	◯	◯	◯
My confidence in my understanding of this statement is:	◯	◯	◯	◯	◯
I think the acceptability of this statement is:	◯	◯	◯	◯	◯

25. Do you have any other comments you would like to add?

[ ]

Category 3: Phrases that compare randomisation to something else (comparative phrases)

26. “Each person is put in a treatment group randomly (like a lottery).” *

	Very Poor	Poor	Neutral	Good	Very Good
My understanding of this statement is:	◯	◯	◯	◯	◯
My confidence in my understanding of this statement is:	◯	◯	◯	◯	◯
I think the acceptability of this statement is:	◯	◯	◯	◯	◯

27. “The choice of what to give (active treatment or dummy treatment) is made randomly (like a lottery)” *

	Very Poor	Poor	Neutral	Good	Very Good
My understanding of this statement is:	○	○	○	○	○
My confidence in my understanding of this statement is:	○	○	○	○	○
I think the acceptability of this statement is:	○	○	○	○	○

28. “People who take part in [study name] are assigned to one of two groups at random (like a lottery)” *

	Very Poor	Poor	Neutral	Good	Very Good
My understanding of this statement is:	○	○	○	○	○
My confidence in my understanding of this statement is:	○	○	○	○	○
I think the acceptability of this statement is:	○	○	○	○	○

29. “If you decide to join the trial, you may receive the investigational treatment chosen by chance, as if on the roll of a dice” *

	Very Poor	Poor	Neutral	Good	Very Good
My understanding of this statement is:	◯	◯	◯	◯	◯
My confidence in my understanding of this statement is:	◯	◯	◯	◯	◯
I think the acceptability of this statement is:	◯	◯	◯	◯	◯

30. “That means that a computer will decide which treatment you have, a bit like rolling a dice for a game” *

	Very Poor	Poor	Neutral	Good	Very Good
My understanding of this statement is:	○	○	○	○	○
My confidence in my understanding of this statement is:	○	○	○	○	○
I think the acceptability of this statement is:	○	○	○	○	○

31. “Patients will be allocated to both groups at random (like tossing a coin)” *

	Very Poor	Poor	Neutral	Good	Very Good
My understanding of this statement is:	○	○	○	○	○
My confidence in my understanding of this statement is:	○	○	○	○	○
I think the acceptability of this statement is:	○	○	○	○	○

32. “You are assigned [study drug] or the dummy drug ‘at random’ (like the toss of a coin) which allows us to properly test whether the [study drug] works.” *

	Very Poor	Poor	Neutral	Good	Very Good
My understanding of this statement is:	○	○	○	○	○
My confidence in my understanding of this statement is:	○	○	○	○	○
I think the acceptability of this statement is:	○	○	○	○	○

33. “Randomisation is similar to tossing a coin to decide which group you are entered into” *

	Very Poor	Poor	Neutral	Good	Very Good
My understanding of this statement is:	○	○	○	○	○
My confidence in my understanding of this statement is:	○	○	○	○	○
I think the acceptability of this statement is:	○	○	○	○	○

34. “You will have an equal chance of being allocated to either group, like the toss of a coin” *

	Very Poor	Poor	Neutral	Good	Very Good
My understanding of this statement is:	○	○	○	○	○
My confidence in my understanding of this statement is:	○	○	○	○	○
I think the acceptability of this statement is:	○	○	○	○	○

35. “The groups are selected by a computer at random, like flipping a coin.” *

	Very Poor	Poor	Neutral	Good	Very Good
My understanding of this statement is:	○	○	○	○	○
My confidence in my understanding of this statement is:	○	○	○	○	○
I think the acceptability of this statement is:	○	○	○	○	○

36. “Whether or not you are given [study drug] or dummy medicine will be decided by chance, rather like tossing a coin to get heads or tails.” *

	Very Poor	Poor	Neutral	Good	Very Good
My understanding of this statement is:	◯	◯	◯	◯	◯
My confidence in my understanding of this statement is:	◯	◯	◯	◯	◯
I think the acceptability of this statement is:	◯	◯	◯	◯	◯

37. Do you have any other comments you would like to add?

Category 4: Phrases that give further details of the ran-domisation (elaborating phrases)

38. “There is a 50/50 chance of being in each group” *

	Very Poor	Poor	Neutral	Good	Very Good
My understanding of this statement is:	○	○	○	○	○
My confidence in my understanding of this statement is:	○	○	○	○	○
I think the acceptability of this statement is:	○	○	○	○	○

39. “You will have a 50% chance of being in either group” *

	Very Poor	Poor	Neutral	Good	Very Good
My understanding of this statement is:	◯	◯	◯	◯	◯
My confidence in my understanding of this statement is:	◯	◯	◯	◯	◯
I think the acceptability of this statement is:	◯	◯	◯	◯	◯

40. “50 % (1 in 2) chance of receiving [study drug] and a 50 % (1 in 2) chance of receiving placebo.” *

	Very Poor	Poor	Neutral	Good	Very Good
My understanding of this statement is:	◯	◯	◯	◯	◯
My confidence in my understanding of this statement is:	◯	◯	◯	◯	◯
I think the acceptability of this statement is:	◯	◯	◯	◯	◯

41. “There is an equal (50:50) chance of being allocated to one group or the other” *

	Very Poor	Poor	Neutral	Good	Very Good
My understanding of this statement is:	◯	◯	◯	◯	◯
My confidence in my understanding of this statement is:	◯	◯	◯	◯	◯
I think the acceptability of this statement is:	◯	◯	◯	◯	◯

42. “You will have an equal chance of receiving the [study drug] treatment or the placebo treatment.” *

	Very Poor	Poor	Neutral	Good	Very Good
My understanding of this statement is:	◯	◯	◯	◯	◯
My confidence in my understanding of this statement is:	◯	◯	◯	◯	◯
I think the acceptability of this statement is:	◯	◯	◯	◯	◯

43. “Half the patients will have [study intervention] and the other half will not” *

	Very Poor	Poor	Neutral	Good	Very Good
My understanding of this statement is:	○	○	○	○	○
My confidence in my understanding of this statement is:	○	○	○	○	○
I think the acceptability of this statement is:	○	○	○	○	○

44. “Half of those taking part in the study will receive [intervention A] and half will receive [intervention B], so you will have a 50% chance of getting either treatment” *

	Very Poor	Poor	Neutral	Good	Very Good
My understanding of this statement is:	○	○	○	○	○
My confidence in my understanding of this statement is:	○	○	○	○	○
I think the acceptability of this statement is:	○	○	○	○	○

45. Do you have any other comments you would like to add?

Category 5: Phrases that describe the process of

46. “Neither you nor your clinical team will be able to decide which study treatment you receive.” *

	Very Poor	Poor	Neutral	Good	Very Good
My understanding of this statement is:	○	○	○	○	○
My confidence in my understanding of this statement is:	○	○	○	○	○
I think the acceptability of this statement is:	○	○	○	○	○

47. “Can I pick which group to go in (i.e. whether to have [the intervention] or not)? No. Neither you nor your surgeon can choose which treatment you receive.” *

	Very Poor	Poor	Neutral	Good	Very Good
My understanding of this statement is:	○	○	○	○	○
My confidence in my understanding of this statement is:	○	○	○	○	○
I think the acceptability of this statement is:	○	○	○	○	○

48. “By taking part in the study you will give up your right to choose which treatment you receive.” *

	Very Poor	Poor	Neutral	Good	Very Good
My understanding of this statement is:	○	○	○	○	○
My confidence in my understanding of this statement is:	○	○	○	○	○
I think the acceptability of this statement is:	○	○	○	○	○

49. “People taking part in [study name] are allocated at random by the [study name] study office to one of two groups” *

	Very Poor	Poor	Neutral	Good	Very Good
My understanding of this statement is:	○	○	○	○	○
My confidence in my understanding of this statement is:	○	○	○	○	○
I think the acceptability of this statement is:	○	○	○	○	○

50. “The decision as to which group patients are assigned to is made at random by the central study office” *

	Very Poor	Poor	Neutral	Good	Very Good
My understanding of this statement is:	○	○	○	○	○
My confidence in my understanding of this statement is:	○	○	○	○	○
I think the acceptability of this statement is:	○	○	○	○	○

51. “Neither you nor your doctor chooses whether you get one of the new drugs but that choice is made by a computer” *

	Very Poor	Poor	Neutral	Good	Very Good
My understanding of this statement is:	◯	◯	◯	◯	◯
My confidence in my understanding of this statement is:	◯	◯	◯	◯	◯
I think the acceptability of this statement is:	◯	◯	◯	◯	◯

52. “If you decide to take part in the trial a computer will decide whether you receive [study drug] or placebo.” *

	Very Poor	Poor	Neutral	Good	Very Good
My understanding of this statement is:	◯	◯	◯	◯	◯
My confidence in my understanding of this statement is:	◯	◯	◯	◯	◯
I think the acceptability of this statement is:	◯	◯	◯	◯	◯

53. “The treatment which you receive will be picked by a computer which has no information about you, that is, by chance.” *

	Very Poor	Poor	Neutral	Good	Very Good
My understanding of this statement is:	○	○	○	○	○
My confidence in my understanding of this statement is:	○	○	○	○	○
I think the acceptability of this statement is:	○	○	○	○	○

54. “In this sort of study, people taking part are put into one of two groups at random by a computer...the researcher will use a computer programme to put you into either Group A or Group B” *

	Very Poor	Poor	Neutral	Good	Very Good
My understanding of this statement is:	○	○	○	○	○
My confidence in my understanding of this statement is:	○	○	○	○	○
I think the acceptability of this statement is:	○	○	○	○	○

55. “That the [group] you (are invited to) join is determined by a sophisticated machine designed for this purpose, and not influenced by us” *

	Very Poor	Poor	Neutral	Good	Very Good
My understanding of this statement is:	○	○	○	○	○
My confidence in my understanding of this statement is:	○	○	○	○	○
I think the acceptability of this statement is:	○	○	○	○	○

56. “The envelopes contain a piece of paper with [intervention A] or [intervention B] written on it and no one knows which method will be used until the envelope is opened.” *

	Very Poor	Poor	Neutral	Good	Very Good
My understanding of this statement is:	◯	◯	◯	◯	◯
My confidence in my understanding of this statement is:	◯	◯	◯	◯	◯
I think the acceptability of this statement is:	◯	◯	◯	◯	◯

57. Do you have any other comments you would like to add?

This content is neither created nor endorsed by Microsoft. The data you submit will be sent to the form owner.

Microsoft Forms
