# Supplementary material for: Trial participants’ self-reported understanding of randomisation phrases in participation information leaflets can be high, but acceptability of some descriptions is low, especially those linked to gambling and luck
Source: Trials. 2024 Jun 18;25:391. doi: 10.1186/s13063-024-08217-3 (PMC11186130; doi:10.1186/s13063-024-08217-3)
Supplement: Supplementary file 2 — Additional file 2: Acceptability, understanding and confidence in understanding scores for each category of phrases. [file 13063_2024_8217_MOESM2_ESM.docx]

Acceptability, Understanding and Confidence in Understanding scores for each category, along with the mean of each category. Category 4, elaborating randomisation phrase, had the highest mean acceptability score. Category 5, phrases that described the process of randomisation had the lowest mean score. The mean acceptability score was always lower than the mean understanding and mean confidence in understanding scores.

**Acceptability, Understanding and confidence in understanding scores for each category of phrases (Proportion of people rating ‘Good or very good’)**

| **Acceptability (A), Understanding (U) & Confidence in Understanding (CU) scores (good + very good) /100** | | | | | | | | | | | | | | | |
| --- | --- | --- | --- | --- | --- | --- | --- | --- | --- | --- | --- | --- | --- | --- | --- |
|  | **CAT 1** | | | **CAT 2** | | | **CAT 3** | | | **CAT 4** | | | **CAT 5** | | |
|  | **A** | **U** | **CU** | **A** | **U** | **CU** | **A** | **U** | **CU** | **A** | **U** | **CU** | **A** | **U** | **CU** |
|  | **80.8** | **87.6** | **90.4** | **71.2** | **86.3** | **89.1** | **69.8** | **85** | **89** | **78.1** | **86.3** | **90.4** | **79.5** | **89** | **90.4** |
|  | **75.3** | **89** | **91.8** | **67.1** | **87.7** | **87.6** | **65.7** | **80.8** | **82.2** | **74** | **87.7** | **89** | **67.1** | **89** | **89** |
|  | **71.2** | **91.7** | **89** | **63.0** | **85** | **86.3** | **60.3** | **79.5** | **84.9** | **72.6** | **86.3** | **90.4** | **60.3** | **78** | **82.1** |
|  | **67.1** | **80.9** | **78.1** | **61.6** | **83.5** | **85** | **53.4** | **75.3** | **79.5** | **72.6** | **87.7** | **90.5** | **58.9** | **74** | **78.1** |
|  | **63.1** | **80.8** | **83.5** | **57.5** | **78.1** | **78.1** | **52.0** | **79.4** | **82.2** | **69.9** | **82.2** | **86.3** | **47.9** | **72.6** | **76.7** |
|  | **61.6** | **82.2** | **86.3** | **54.8** | **78** | **82.2** | **50.7** | **74** | **79.5** | **59.9** | **84.9** | **89.1** | **45.2** | **71.3** | **76.7** |
|  | **53.5** | **72.6** | **68.5** | **49.4** | **75.3** | **76.7** | **46.5** | **72.6** | **76.7** | **50.7** | **73.9** | **79.4** | **45.2** | **68.5** | **71.2** |
|  | **50.7** | **69.9** | **67.2** | **41.1** | **71.3** | **69.9** | **43.8** | **80.8** | **82.2** |  |  |  | **38.3** | **64.4** | **72.6** |
|  | **46.6** | **65.8** | **65.7** | **39.5** | **68.5** | **72.6** | **43.8** | **75.4** | **79.4** |  |  |  | **37.0** | **64.4** | **68.5** |
|  | **45.2** | **67.1** | **69.8** |  |  |  | **38.3** | **72.6** | **57.3** |  |  |  | **36.9** | **63** | **69.9** |
|  |  |  |  |  |  |  | **24.7** | **58.9** | **63** |  |  |  | **24.6** | **64.4** | **72.6** |
| **Total** | **570.1** | **787.6** | **790.3** | **468.2** | **713.7** | **727.5** | **549.1** | **834.3** | **855.9** | **477.8** | **589** | **615.1** | **540.9** | **798.6** | **847.8** |
| **Mean** | **57** | **78.8** | **79** | **52** | **79.3** | **80.8** | **49.92** | **75.8** | **77.8** | **68.23** | **84** | **87.9** | **49.17** | **72.6** | **77** |

| **Category** | **Theme** | **Number (n = 48)** |
| --- | --- | --- |
| 1 | Explanation of why randomisation is required in clinical trials. | 10 |
| 2 | Randomisation synonyms (Phrases used to describe randomisation – using different words that mean randomisation). | 9 |
| 3 | Comparative phrases (Phrases that compare randomisation to something else). | 11 |
| 4 | Elaborating Phrases (Phrases that give further details of the randomisation). | 7 |
| 5 | Phrases that describe the process of randomisation. | 11 |
