# Supplementary material for: Trial participants’ self-reported understanding of randomisation phrases in participation information leaflets can be high, but acceptability of some descriptions is low, especially those linked to gambling and luck
Source: Trials. 2024 Jun 18;25:391. doi: 10.1186/s13063-024-08217-3 (PMC11186130; doi:10.1186/s13063-024-08217-3)
Supplement: Supplementary file 3 — Additional file 3: Top 10 randomisation phrases ranked by acceptability. [file 13063_2024_8217_MOESM3_ESM.docx]

**Supplementary File 3: Top 10 randomisation phrases ranked by acceptability.**

| **Top 11 Phrases** | **Phrase with high acceptability** | **Inductive Category to which phrase belongs** | **Understanding (%)** | **Confidence in understanding (%)** | **Acceptability (%)** |
| --- | --- | --- | --- | --- | --- |
| **1** | **“We do randomised trials when there is more than one treatment option available for patients with a disease and we don’t know which one is best. In order to find out, we need to compare the different treatments. So we put people into groups and give each group a different treatment. The results from the different treatment groups are compared to see if one treatment is better. To try to make sure the groups are the same to start with, each patient is put into a group by chance (randomly).”** | Category 1: Why randomisation required | 90.4 | 87.6 | 80.8 |
| **2** | **“Neither you nor your clinical team will be able to decide which study treatment you receive.”** | Category 5: Process of randomisation | 90.4 | 89 | 79.5 |
| **3** | **“There is a equal (50:50) chance of being allocated to one group or the other”.** | Category 4: Elaborating randomisation phrases | 90.4 | 86.3 | 78.1 |
| **4** | **“When we do not know which way of treating patients is best, we need to make a comparison. An important part of making a fair comparison is “randomisation”. Most large trials are randomised. Patients taking part are randomly allocated either the standard treatment or the research treatment. This process is essential to avoid bias: if the groups receiving each treatment are the same, any differences in the results can only be down to the treatments. Therefore, randomisation means that the results are more reliable.”** | Category 1: Why randomisation required | 91.8 | 89 | 75.3 |
| **5** | **“You will have a 50% chance of being in either group”.** | Category 4: Elaborating randomisation phrases | 89 | 87.7 | 74 |
| **6** | **“You will have an equal chance of receiving the [study drug] treatment or the placebo treatment.”** | Category 4: Elaborating randomisation phrases | 90.5 | 87.7 | 72.6 |
|  | **“There is a 50/50 chance of being in each group”.** | Category 4: Elaborating randomisation phrases | 90.4 | 86.3 | 72.6 |
| **8** | **“It is really important that the two groups for the [study name] study have a similar mix of patients in them. Having a similar mix means that we know that if one group of patients does better than the other, it is very likely to be because of the treatment and not because there are differences in the types of patients in each group”.** | Category 1: Why randomisation required | 89 | 91.7 | 71.2 |
|  | **“The decision of which of the two treatments you receive will be made by chance (this is called ‘randomisation’).”** | Category 2: Randomisation synonyms (Phrases used to describe randomisation – using different words that mean randomisation). | 89.1 | 86.3 | 71.2 |
| **10** | **“Half of those taking part in the study will receive [intervention A] and half will receive [intervention B], so you will have a 50% chance of getting either treatment”.** | Category 4: Elaborating randomisation phrases. | 86.3 | 82.2 | 69.9 |
